# Review of Approaches to Minimise the Cost of Simulation-Based Optimisation for Liquid Composite Moulding Processes

**DOI:** 10.3390/ma16247580

**Published:** 2023-12-09

**Authors:** Boon Xian Chai, Boris Eisenbart, Mostafa Nikzad, Bronwyn Fox, Yuqi Wang, Kyaw Hlaing Bwar, Kaiyu Zhang

**Affiliations:** 1 Faculty of Science, Engineering and Technology, Swinburne University of Technology, Hawthorn, VIC 3122, Australia; bchai@swin.edu.au (B.X.C.); yuqiwang@swin.edu.au (Y.W.);; 2 CSIRO Clayton, Clayton, VIC 3168, Australia

**Keywords:** simulation, computational cost, optimisation, composite, liquid composite moulding

## Abstract

The utilisation of numerical process simulation has greatly facilitated the challenging task of liquid composite moulding (LCM) process optimisation, providing ease of solution evaluation at a significantly reduced cost compared to complete reliance on physical prototyping. However, due to the process complexity, such process simulation is still considerably expensive at present. In this paper, cost-saving approaches to minimising the computational cost of simulation-based optimisation for LCM processes are compiled and discussed. Their specific applicability, efficacy, and suitability for various optimisation/moulding scenarios are extensively explored in detail. The comprehensive analysation and assimilation of their operation alongside applicability for the problem domain of interest are accomplished in this paper to further complement and contribute to future simulation-based optimisation capabilities for composite moulding processes. The importance of balancing the cost-accuracy trade-off is also repeatedly emphasised, allowing for substantial cost reductions while ensuring a desirable level of optimization reliability.

## 1. Introduction

Currently, large-scale composite manufacturing is commonly achieved via liquid composite moulding processes [1,2,3,4]. The utilisation of numerical process simulation has greatly facilitated the challenging task of LCM process optimisation, providing ease of solution evaluation at a significantly reduced cost compared to complete reliance on physical prototyping. Nevertheless, the computational cost of performing such composite moulding simulations is still considerably expensive at present, given its complexity [5,6,7]. As a consequence, the overall computational cost of simulation-based optimisation can be enormous, as each solution evaluation is essentially a numerical simulation run that typically requires a long computing time. In fact, within the setting of simulation-based optimisation, the cost of computing the process simulation accounts for a major portion of the total optimisation cost associated. Full-scale numerical simulation of the mould-filling process can become progressively cost-prohibitive to compute as the number of optimisation iterations required increases. Moreover, as more sophisticated and accurate *multi-scale coupled textile-flow models* are progressively being developed, the cost of simulation will further increase in the future, likely by a significant margin compared to contemporary meso-scale *Darcy’s-Law-based flow models* [6,8,9]. Therefore, the high cost of process simulation, in terms of both the computational power and computation time required, needs to be addressed promptly as the key bottleneck to the application of simulation-based optimisation. Principally, the high cost of simulation-based optimisation can be effectively addressed by either: (i) reducing the total number of solution evaluations required during the optimisation process, and/or, (ii) reducing the computational cost of the process simulation [7,10,11,12]. This paper focuses mainly on the latter.

As highlighted in [6,8], the felicitous selection of optimisation algorithms with respect to the problem context (i.e., mould-filling scenario) can greatly reduce the number of solution evaluations required during the optimisation process. Aside from the appropriate selection of optimisation algorithms, how one can effectively utilise problem-specific knowledge and information to streamline the optimisation framework also receives massive attention in the research community [13]. The inclusion and exploitation of known problem structures and characteristics, process constraints, and insights of the mould-filling process during algorithm development and implementation can significantly lessen the resultant optimisation cost. As an extension, depending on the problem context, deliberate contrivances and shortcuts can be employed to circumvent the prohibitively high cost of simulation-based optimisation. Hence, in this paper, cost-saving contrivances and cheaper modelling/computational alternatives tailored for LCM mould-configuration optimisation problems are compiled, with their specific applicability, efficacy, and suitability towards various optimisation/moulding scenarios being explored in detail.

## 2. Parallel Computing

**Figure 1:**
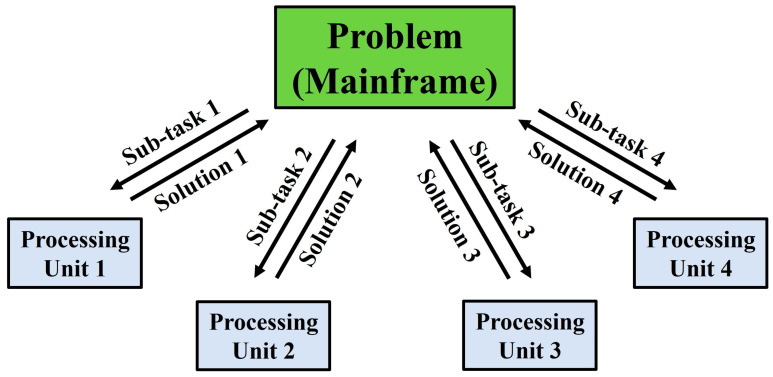
The schematic framework of the application of parallel computing to solve a problem.

In the recent past, computer algorithms have conventionally been developed for serial computing [14,15,16,17]. Consequently, when solving a problem, only a single task (or instruction) is executed at any moment in time. As a result, there is an inefficient utilisation of the hardware resources available, where only a part of the potential computing capability is employed at any particular instance. Nowadays, these superannuated approaches to algorithm design are being progressively phased out as developments in parallel hardware architecture progress steadily [14,17,18]. Thanks to the rapid advancements in the field of parallel computing, a significant portion of the problems faced by serial computing are gradually becoming obsolete. In both academic and industrial settings, the technique of parallel computing is often employed by researchers and industry practitioners alike to hasten the simulation-based optimisation processes [7,10,19,20]. The streamlining of the simulation-based optimisation process via parallel computing for LCM process optimisation is no exception [7,20,21]. Parallel computing can be understood as the act of breaking down a larger, complex problem into numerous smaller, independent sub-tasks and computing them simultaneously across multiple processing units. The individual outputs of these parallel sub-tasks can then be remerged, upon their completion, back into the original problem framework for completion or further analysis. The schematic framework of parallel computing is depicted in Figure 1.

Parallel computing offers several advantages over conventional serial computing. The apportion of a complex problem into multiple independent sub-tasks allows the total computational load to be distributed (either evenly or unevenly) across all available processing units to be computed in parallel simultaneously. Therefore, the undesirable wastage of unutilised or underutilised (*idle*) computing power can be minimised, which is particularly critical in the modern era where multi-core processors are progressively becoming the norm. In addition to the effective distribution and utilisation of computing power, parallel computing also enables the effective employment of non-local resources (e.g., on a wide area network or over the internet) when the local resources are inadequate. Larger problems, too large to fit into a single machine’s memory, can alternatively be solved via parallel computing, thus alleviating hardware constraints while introducing a massive scale-up of computational potential compared to that of local serial computing.

Most importantly, parallel computing allows parallelisable algorithms and applications to be computed within a shorter wall-clock time than serial computing (i.e., faster algorithm execution). While the total computational load remains unchanged, independent computing tasks can be distributed across multiple processors or computing machines, drastically compressing the computing time required from start to finish [15,17,21,22]. The computational time saving is commonly quantified by the *speedup*, which is defined as the proportion of the cost of solving a parallelisable problem/algorithm via a single processing unit versus that of solving it parallelly across multiple processing units. In the context of minimising the cost of simulation-based optimisation problems, studies across the literature have reported appealing cost reductions ranging around the range of 65% to as high as 92% [8,22,23,24]. The reduction in computing time attained via parallel computing effectively accelerates the respective project timeline and compresses the corresponding time to market, giving the users a competitive edge over their competitors. Last, but not least, parallel computing helps facilitate real-time updating and monitoring of the process progression while the upcoming computations are performing in the background, bringing concurrency and flexibility to its users [7,14,17,25].

There are many strategies for implementing parallel computing in simulation-based optimisation settings, with their selection dependent on the problem at hand. It is worth noting that the implementation of parallel computing is, to a certain extent, restricted by the (parallel) hardware architectures alongside that of the algorithm. With respect to the state-of-the-art technological advancements to date, parallel computing can be executed on multitudes of parallel architecture hierarchies, ranging from a single computer equipped with multiple processing units (CPUs, GPUs, cores) to cloud computing and computer clusters (or grids) that host multiple network-connected stand-alone computers [10,14,18,22]. Currently, there are four broad types of parallelism achievable in parallel computing, namely: bit-level parallelism, instruction-level parallelism, task parallelism, and data-level parallelism [14,17,18]. The topic of interest here, which is the cost reduction of simulation-based optimisation via parallel computing, mainly pertains to task parallelism and data-level parallelism. When performing the simulation-based optimisation, optimisation algorithms that can execute the search process without requiring knowledge of prior solution evaluations can be parallelised for parallel computing. This generally pertains to algorithms that attempt to solve the optimisation problem by brute force, with some examples including the exhaustive search and unguided random search. For this kind of algorithm, the adoption of parallel computing will potentially lead to a maximum theoretical speedup $S_{THEORETICAL}$ proportionate to *N*:

(1) $$Theoretical\;speedup,S_{THEORETICAL}\propto N$$

where *N* can be either:

(i) the number of processing units, or
(ii) the size of the problem, depending on the hardware’s parallel architecture. Note, only a minor proportion of all contemporary algorithms can be decomposed into completely independent pieces, enabling the theoretical linear speedup.

**Figure 2:**
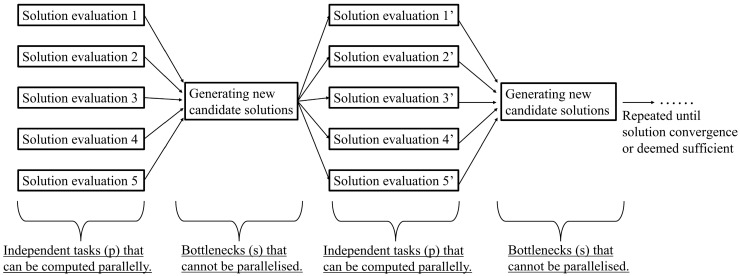
The generic search flow diagram of population-based optimisation algorithms.

Besides that, the inherent parallelism of population-based algorithms (i.e., evolutionary algorithms) can also be exploited. This is so as population-based algorithms typically consider or evaluate multiple candidate solutions collectively prior to each impending search phase, as depicted in Figure 2. Effective parallelisation is thus possible as the outputs of the solution evaluation of each candidate solution are distinct from each other, allowing them to be computed independently. Some notable examples include the genetic algorithm, ant colony optimisation, and particle swarm optimisation.

By performing the independent solution evaluations simultaneously, the search process of the population-based optimisation algorithms can be expedited significantly. Do note, the maximum performance improvement achievable is limited by the fraction of parallelisable components within the population-based optimisation algorithms. The theoretical speedup $S_{THEORETICAL}$ for the population-based algorithms by parallel computing can be expressed by the Amdahl’s law [14,15,17,22], as:

(2) $$Theoretical\;speedup,\;S_{THEORETICAL}\leq \frac{1}{(1-P)+\frac{P}{N}}$$

where *P* is the fraction of the independent tasks within the algorithm that can be executed parallelly (e.g., evaluating the individuals within each generation of GA) and *N* is the number of processing units utilised.

Parallel computing can also be adopted to minimise the computational cost of the statistical modelling and characterisation for LCM processes via the Monte Carlo simulation approach [8,14,22]. These statistical analyses are critical to combat the issues of process randomness and lack of process repeatability within the LCM processes [8,26]. Parallel computing allows the user to perform the parallel computation of stochastic simulations for statistical modelling purposes and to perform parallel replications of a stochastic simulation for statistical characterisation purposes. Minimising the computational cost of these stochastic simulations will aid in securing the process robustness of the mould-filling stage [7,8,27]. Additionally, parallel computing can also be extremely valuable for the development and training of metamodels as the metamodel training data required are generally independent of one another, allowing parallelism [7,8,14].

**Figure 3:**
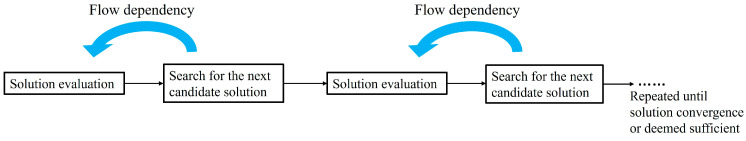
The generic search flow diagram of single-solution serial algorithms.

While there are many levels of parallelism attainable, not every optimisation algorithm can exploit the merits of parallel computing in the setting of simulation-based optimisation. The adoption of certain algorithm structures, which is often dictated by the nature of the problem itself, may prohibit the simultaneous execution of computing tasks and prevent effective parallelisation [8,14,17,24]. Moreover, the issue of flow dependency is also pertinent to the adoption of parallel computing in simulation-based optimisation. Flow dependency, also commonly known as *read-after-write* (*RAW*), refers to the scenario where the execution of a task is dependent on the output of its preceding task [14,15,17,24]. As such, parallel computing is practically ineffectual for single-solution serial optimisation algorithms that: (i) evaluate only a single candidate solution during each evaluation iteration; and (ii) require knowledge of prior solution evaluation(s) to guide the following search phase (i.e., the *exploration/search* mechanism). For this type of algorithm, as each search phase is dependent on the result of its prior solution evaluation(s), the upcoming search tasks are forced to remain on hold until the prior solution evaluation is completed, preventing the effective distribution of computational workload. The generic search flow diagram of single-solution serial algorithms is depicted in Figure 3.

In summary, while the adoption of parallel computing has great potential in the application of simulation-based optimisation, its efficacy and applicability are highly dependent on the degree of achievable parallelism imposed by the algorithm’s framework and its flow dependencies [8,14,17,21]. Besides that, the application of parallel computing requires the development and execution of additional auxiliary algorithms to parallelise the existing optimisation framework (e.g., for task partitioning, task scheduling, task synchronisation, etc.) [14,17,19,22]. Lastly, the framework of parallel computing can be challenging to construct and implement. The complex operations of data transfer, memory organisation, communication, and synchronisation between multiple (locally or non-locally) independent processing units may require a significant effort to maintain smoothly [14,15,16,17]. In particular, issues arising from network latency and the non-homogeneity in computational power across the independent processing units can greatly complicate the vital tasks of communication and synchronisation during parallel computing. The overhead cost of these control operations can also be a deterrent to the adoption of parallel computing, as these fundamental operations can be computationally demanding to execute as well [14,16,17,19]. A delicate trade-off between the additional computational cost required versus the computational time saved is thus essential for the effective application of parallel computing in simulation-based optimisation settings.

## 3. Time Integration for Numerical Simulation Computation

Generally, the mould-filling phenomenon of the LCM processes is numerically simulated by either the finite elements/control volume approach or the non-conforming finite elements approach, both of which are numerical techniques for solving the governing differential equations (ODEs, PDEs) on a discretised (space and time) domain [11,28,29]. The governing equations for the pressure field and the velocity field can be obtained by integrating Darcy’s law into the mass continuity equation. As the temporal evolution of the process is of interest, time discretisation is implemented, by dividing the investigated process intervals into a set of multiple short time-steps. The transient mould-filling process is often treated in the finite elements (FE) approaches as a quasi-static/quasi-steady process, assuming a steady state is achieved at each time step throughout the temporal integration [28,30,31,32]. The numerical analysis can then be computed by either the *explicit time-integration scheme* or the *implicit time-integration scheme* to obtain the numerical approximations of the mould-filling process. The selection of which time-integration scheme to adopt is critical to the numerical analysis’ stability, efficiency, and accuracy, which in turn affect the cost and performance of the proceeding simulation-based optimisation [17,30,31,33].

While both time-integration schemes operate on a similar numerical approximation mechanism, they differ in the selections of the spatial/process derivatives and the time incrementation during the numerical computation. To clearly portray the difference between the explicit and implicit time-integration schemes, assume a simple system of ODE (e.g., one dimensional resin flow) such that:

(3) $$\frac{dv}{dt}=f\left(v\right)$$

where v is a process-dependent vector (e.g., resin velocity) and $f\left(v\right)\;$is the process governing function (e.g., Darcy’s law/mass continuity equation). During the numerical analysis, the explicit time-integration scheme (also known as the *forward Euler method*) evaluates the governing function $f\left(v\right)$ at a current time to predict the resultant future state. Hence, starting from an initial (known) process state *n*, to numerically predict a future (unknown) process state *n +* 1, the mathematical formulation of the explicit scheme will take on the form of:

(4) $$v_{n+1}=v_{n}+∆t\cdot f(v_{n})$$

On the contrary, during the numerical analysis, the implicit time-integration scheme (also known as the *backward Euler method*) evaluates the governing function $f\left(v\right)$ at a future time to predict the corresponding future state. As such, starting from an initial (known) process state *n*, to numerically predict a future (unknown) process state *n +* 1, the mathematical formulation of the implicit scheme will take on the form of:

(5) $$v_{n+1}=v_{n}+∆t\cdot f(v_{n+1})$$

To recap, the explicit scheme predicts the future process state from the current process state while the implicit scheme predicts the future process state from both the current and the future process states.

For the explicit time-integration scheme, since both the current process state $v_{n}$ and the current state’s process derivative $f(v_{n})$ are known, its individual time-step calculation is straightforward and not costly to compute. However, the maximum time-step size possible for the explicit time-integration scheme is bounded by the stability limit defined by the Courant–Friedrichs–Lewy condition [30,31,33,34]. The time-step size chosen needs to be small enough to ensure that the output solution is stable and divergence-free due to the extrapolation nature of the explicit time-integration scheme (*conditionally stable*). Since the current process state and derivative are used to predict that of the future, extrapolating too far into the unknown future state will introduce errors into the numerical predictions. These rounding errors could be cumulatively magnified across the temporal evolution, causing the output to be unbounded, leading to numerical instability. Overall, although its individual time-step calculation is not costly, the explicit time-integration scheme may become exponentially more expensive if too many time steps are required, especially if the model to be numerically evaluated is huge.

For the implicit time-integration scheme, only the current process state $v_{n}$ is known, while the future state’s process derivative $f(v_{n+1})$ is unknown and needs to be solved. The unknown future derivative is typically solved by either matrix inversion or by the Newton–Raphson method (*iterative root-finding algorithm*). Due to the need for non-causal recursive computation, the implicit time-integration scheme is more computationally expensive to solve at each time step compared to that of the explicit scheme. Notably, since global equilibrium is achieved in each time step, there is no solution stability (*divergence*) concern for the implicit time-integration scheme [31,35,36,37]. Hence, larger time-step sizes can be adopted by the implicit time-integration scheme since it is not bounded by any stability conditions or limitations (*unconditionally stable*). If the time-step size adopted is large enough, the overall computational cost of the implicit time-integration can be significantly less than that of the explicit scheme. Nonetheless, the implicit time-integration scheme may still be inaccurate if the time-step size chosen is unproportionately large with respect to the phenomenon evaluated [21,31,33,38].

By avoiding the need for non-causal recursive computations and convergence at each time step, the explicit time-integration scheme is typically adopted for highly non-linear problems with a high number of degrees of freedom (DOF), where time-step calculations are consequential and tend to diverge [31,33,34,38]. The explicit time-integration scheme is generally recommended for fast-moving, short-duration dynamics analyses, such as impact phenomena that usually occur within milliseconds to seconds. As the total process durations of such analyses are typically short, the computational cost of the explicit scheme remains practicable even when adopting a very small time-step size. On the other hand, the implicit time-integration scheme is able to accommodate larger time-step sizes with no stability concerns. As such, the implicit time-integration scheme is more suited for slower dynamics processes with fewer non-linearities, such as the mould-filling phenomenon of the LCM processes, which could last around minutes to hours with relatively stable process conditions [32,33,35,39]. The implicit time-integration scheme is also often preferred as many problems in practice are stiff (*numerically unstable*), whereas the explicit scheme will require an impractically small time-step size to bound the error in the results. For the same process duration and desired accuracy, the explicit scheme’s adoption of smaller time steps will result in an enormous amount of iterations required, which could be cheaper to solve by the implicit scheme with larger, albeit more costly, time steps [7,17,21,30].

**Figure 4:**
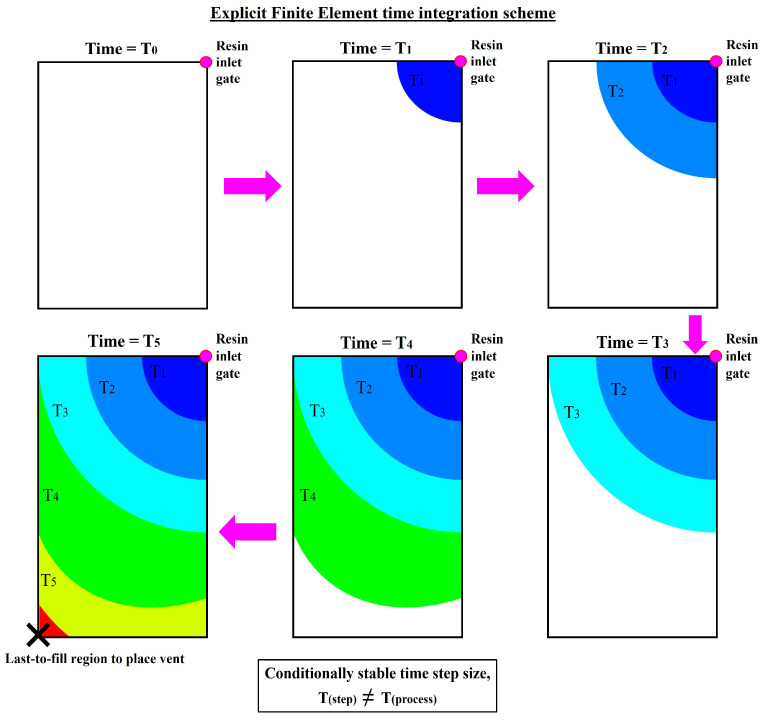
An example of the temporal evolution of a mould-filling simulation adopting the explicit time-integration scheme.

**Figure 5:**
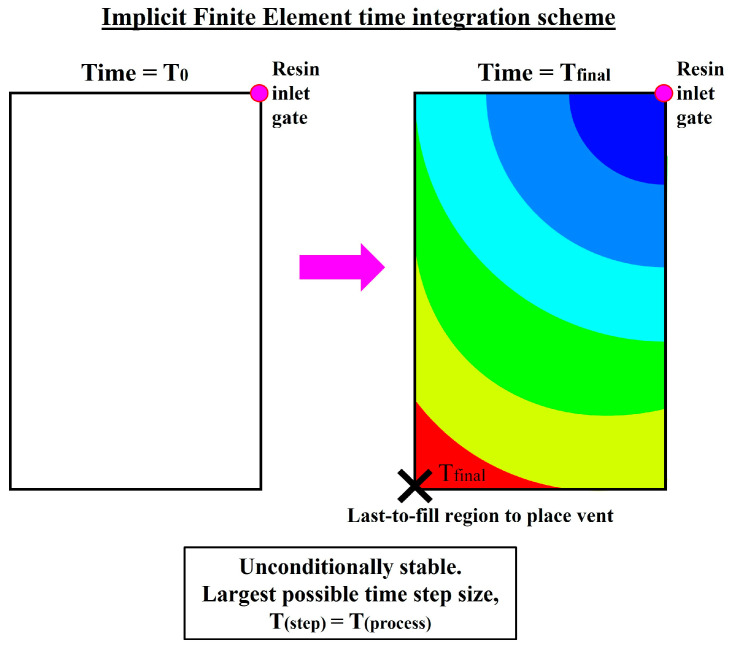
An example of the one-shot computation of a mould-filling simulation adopting the implicit time-integration scheme.

For simulation-based optimisation applications, the main cost distinction between the explicit and implicit time-integration schemes arises primarily from their selection of the time-step size. Using the RTM mould-filling simulation as an illustration, the time-step size chosen by the explicit time-integration scheme needs to be associated to one steady flow, where no more than one control volume within the discretised domain becomes filled at each time step [25,31,40]. The time to fill each subsequent control volume can be computed from the following relation:

(6) $$∆t=\frac{(1-f)V}{\sum Q}$$

where ∆t is the time-step size, f is the fill factor, V is the volume of the control volume, and Q is the volumetric flow rate in the control volume. Ergo, the explicit time-integration scheme iteratively evaluates the mould-filling progression via a series of small time steps which cumulatively sum up to the total process duration, as schematically depicted in Figure 4. Conversely, the implicit time-integration scheme can numerically predict the future process states via any large time steps, which are not restricted to just one control volume-steady flow [31,33,39,41]. By adopting larger time-step sizes, lesser number of time steps are required to numerically simulate the mould-filling process from start to finish. In fact, for simple problems (e.g., non-varying, low DOF problems), the largest possible time-step size can theoretically be as large as the total process time itself (i.e., *T_step_ = T_process_*), solving such problems in just a single time step [31,35,37,39], as schematically depicted in Figure 5.

In optimisation scenarios where the primary research interest lies not on the filling progression but rather on the final state of the mould-filling process, computing the entire mould-filling simulation from start to finish via the explicit scheme-based multi-time-step numerical computation is often uneconomical and cost-prohibitive [8,21,37,41]. For instance, when dealing with the optimisation of the vent locations on the mould (e.g., in the presence of stochastic disturbances), the progression of the resin flow is not of as much of a significance compared to the identification of the last-to-fill areas within the mould [8,21,35,37]. To deal with simulation-based optimisation problems of such a nature, researchers proposed the adoption of the implicit time-integration scheme to numerically predict the end-of-filling state of LCM processes. Voller et al. [30,35] first proposed a finite element/control volume implicit algorithm to mimic the fluid flow in porous media, which was then further refined by Lin et al. [39] and Chen et al. [37] to simulate the 2D and 2.5D (thin-shell) resin permeation in the RTM process. Extensions to 3D RTM mould filling are achieved by Mohan et al. [33] using a pure finite element approach. Given the initial boundary conditions (e.g., resin injection pressure) and the non-varying process conditions (e.g., reinforcement permeability), these proposed implicit algorithms operate by invoking and coupling the mould-filling governing equations with the filled volume fractions to determine the solved domain, which is based on the summability and integrability of the governing equations [30,31,33,39]. The implicit computation of the evolving mould-filling pattern, albeit more costly to solve, effectively removes the stability restriction on the time steps. The time-step size is then only dependent on the time resolution and the desired simulation accuracy [30,33,37,39].

Nevertheless, the adoption of the implicit time-integration scheme to numerically simulate the LCM mould-filling phenomenon is accompanied by multiple assumptions and prerequisites [30,33,35,39]. Firstly, the mould-filling process needs to be relatively linear with non-varying boundary and filling conditions (i.e., isothermal filling with no resin curing). In addition, the mould is to be modelled as a rigid body with a fixed mould volume, implying that LCM processes utilising flexible moulds such as the RTM-light and RI processes could not be simulated implicitly. Also, due to the coupling of Darcy’s law with the continuity equation and the filled-fraction field, only the filling of reinforcements with a homogeneous permeability profile (which can be either isotropic or anisotropic) can be computed. While the phenomenon of race-tracking can be introduced, an additional accompanying assumption is necessary: air pushed by the moving resin is assumed to be able to escape from the mould boundaries, which can only happen if a peripheral vent design is adopted. Under these assumptions, for 1D, 2D, and 2.5D mould-filling scenarios, the implicit algorithms proposed in the literature are capable of *one-shot* (i.e., in a single time step) predicting the last-to-fill locations within the mould and the potential air-trap locations by identifying the local minima within the computed pressure field [30,37,39]. For 3D mould-filling scenarios, the implicit scheme proposed by Mohan [33] is incapable of predicting the end-of-filling state in just a single time step. That said, the implicit algorithm could still numerically evaluate the filling process in large time steps, maintaining credible solution accuracy while concurrently saving valuable computational resources [31,33,38].

The felicitous selection of the time-integration scheme during the numerical computation plays a crucial role in conserving the overall computational cost of simulation-based optimisation. This is particularly true for the adoption of the implicit time-integration scheme to simulate the mould-filling phenomenon during the LCM processes, especially in problems where only the end-of-filling state is of interest. Some notable examples include the optimisation of the vent locations, mould-filling optimisation via the Monte-Carlo methods, and on-line (active) controlled mould-filling processes. Cross-validated with the multi-time-step explicit scheme-based simulations and some known analytical (closed-form) solutions, researchers have demonstrated that the implicit schemes proposed can reliably and accurately predict the final filling state of LCM mould-filling processes [30,31,33,39]. Using an implicit time-step size 10 to 100 times larger than that of the explicit time-step size on various large, thin-shelled composite models, Jiang & Duan [31] and Minaie & Chen [36] reported computation savings of up to 60%, while Lin et al. [39] reported a range of 53–71% in computational savings. For 3D mould-filling scenarios, Mohan et al. [33] reported a range of 85–92% in computational savings.

Note, while the formulation of the implicit scheme is unconditionally stable and can handle any large time-step size, its convergence and stability do not always equate to accuracy. The implicit scheme’s time resolution and solution accuracy are still predominantly dependent on the time-step size chosen. Adopting a time-step size too large for the problem at hand (e.g., a time-step size of >10 min for simplified flow problems) will lead to a potential under- or over-estimation of the numerical predictions [33,37,38,39]. It is also vital to highlight that the adoption of the implicit time-integration scheme is accompanied by its many prohibitory assumptions and prerequisites. As mentioned earlier, the implicit computation’s formulation is constructed on the foundation of non-varying process/boundary conditions and the homogeneity in the reinforcement permeability. While the prerequisite of having non-varying process/boundary conditions can be attained easily (e.g., with the addition of curing inhibitors), its restricted applicability to only reinforcements of homogeneous permeability profiles greatly hinders its adoption in the industry. As the introduction of composite components increases across various industries, their mechanical and structural requirements are becoming more tailored and specific. This gives rise to the necessity to introduce inserts into preforms or form preforms of inhomogeneous permeability in an effort to satisfy the parts’ performance requirements [38,42,43]. As a consequence, the adoption of preforms with a homogeneous permeability profile is being progressively phased out. In such manufacturing scenarios, the implicit time-integration scheme will struggle to compute an accurate pressure/velocity governing field to simulate the mould-filling process.

## 4. Problem Transformation

During a typical optimisation process, the problem domain, design space, and problem landscape regularly take on a convoluted and unintuitive form [7,44,45,46]. As a result, effective human intervention and analysis during the optimisation process are often hindered, especially for the vital tasks of problem analysis and decision making [7,12,45,47]. Researchers have thus proposed the concept of problem transformation, where the original optimisation problem is translated into another optimisation problem of a different form. The simplified, generic formulation of problem transformation is provided as follows. Without loss of generality, let *Z* be a minimisation problem with *n* decision variables and *m* optimisation objectives. For the problem of LCM injection configuration optimisation, the decision variables are the gate and vent locations on the mould, while the objectives *f* are generally some yield-/cost-driven process outputs such as the mould-filling time required. The optimisation problem can thus be expressed in mathematical notation as:

(7) Z :min ⁡Fx=fx for single-objective optimisation

Or

(8) Z :min ⁡Fx=(f1x, f2x, …, fmx) for multi-objective optimisation

$$s.t.\;x\in \Omega \subseteq R^{n}$$

with Ω denoting the search space (i.e., all potential gate nodes lying on the mould geometry). The optimisation aim is then: find at least one point x* satisfying *f*(x*) ≤ *f*(*x*) for all *x* ∈ R. For any solution vector $\vec{x}$, $f(\vec{x})$ can be rewritten as: $f(\psi \left(\vec{w},\vec{x}\right))$, with a vector $\vec{w}$ derived from *x* such that [8,16,46,48]:

(9) $$\forall x_{i}\;:\exists w_{i}\;:w_{i}x_{i}=x_{i}^{*}$$

(10) $$\forall \vec{x}\in R^{n}\;:\exists \vec{w}\;:\psi \left(\vec{w},\vec{x}\right)={\vec{x}}^{*}$$

Using a problem-domain transformation function ψ, the original optimisation problem Z can then be reformulated into a new problem Z* which optimises the vector $\vec{w}$ such that:

(11) $$Z^{*}\;:\min\;F\left(\vec{w}\right)=(f_{1}\left(\vec{w}\right),f_{2}\left(\vec{w}\right),\ldots ,f_{m}\left(\vec{w}\right))$$

$$s.t.\;\vec{w}\in \varphi \subseteq R^{n}$$

with φ denoting the transformed search-space state. The domain transformation function ψ defines how the movement takes place inside this space. Generally, a permutation of Ω (original search space) to φ (transformed search space) is a bijective (i.e., *one-to-one*) mapping from Z to Z*, though exceptions exist [16,46,47,49].

**Figure 6:**
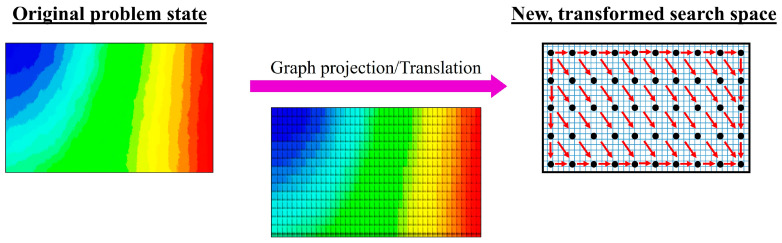
A generic graphical example of the application of search-space (domain) transformation.

Problem transformation is typically achieved by projecting or translating the search space onto a graphical plane, which transforms and simplifies the current problem domain into a new search-space state [16,44,47,50], as depicted in Figure 6. In fact, a majority of optimisation problems can be defined naturally and expressed by means of graphs [8,42,47,51]. A graph, which is often used as an abstract representation of models, can be understood as a structure amounting to a set of interdependent objects represented as edges and vertices; an edge is a link between two vertices. Geometric graph structures (e.g., weighted graph, directed graph) are frequently adopted due to the fact that their graphical structure eases algorithm integration and implementation while concurrently enabling effective visualisation and interpretation of the problem at hand [7,45,47,51]. Veritably, search-space transformation is crucial for the application of graph-search algorithms on non-graph-based optimisation problems [8,16,47,51]. The major impetus of problem transformation is akin to that of heuristics: by exploiting known process knowledge and regularities, the optimisation problem can be simplified and solved in a more efficient manner [13,16,47,52]. The concept of problem transformation relies heavily on the exploitation of the underlying geometrical features and problem simplification [10,48,53]. Thus, in-depth knowledge of the problem domain and search-space structure are required of the practitioner for the effective application of problem transformation [8,13,53].

The concept of problem (search-space) transformation has gained significant research attention, boasting the attractive merits of hastening search convergence, decreasing the risk of local stagnation, and improving solution quality [44,46,50,51]. There are also studies suggesting that the felicitous application of problem transformation can significantly reduce the search-space size, highlight search-space regularities, and aid in finding the hidden/implicit relationships between the solutions [8,16,53]. The transformed space also acts as an intuitive diagrammatic representation of the problem domain and design space, which could assist the practitioner with making informed decisions during the early stages of process development/optimisation [7,45,47,54]. The graphical translation of the search space is also reported to be instrumental for the identification of suitable search starting points within the search space for the optimisation operation, which can be extremely helpful when dealing with problems involving a large search space (e.g., large moulds containing a large number of potential gate/vent nodes) [8,42,43,54].

For the optimisation of mould configuration for LCM processes, the search space, which is formed by all of the potential gate/vent nodes on the mesh bounded by the mould geometry, can be transformed into a graph state by mapping critical process information such as the pressure profile within the mould during mould filling [27,51] or the resin progression (flow) pattern [42,54] to a graph defined by desired cost functional(s). Various space-partitioning computational geometry models such as the geometric-based BBS [53], the medial axis [42,54], and the centroidal Voronoi tessellation (CVT) [38,55] can also be plotted onto a graph plane to facilitate the optimisation process. From a mathematical viewpoint, the LCM mould-configuration optimisation problem can then be treated as a *graph theory–mathematical optimisation* problem [8,47,48,51]. Upon transforming the search space into a graph structure, efficient graph-search algorithms can then be introduced to solve the optimisation problem using information of the graph structure, such as the geodesic distance between the graph’s vertices. Some notable problem transformation examples in the literature are provided in the following. Ye et al. [51] and Li et al. [27] innovatively transformed the RTM injection configuration optimisation problem into the *shortest path problem* by capitalising on the strong positive correlation between the resin flow distance and the mould-filling time. The search space is first transformed into a directed weighted graph consisting of vertices (denoting the candidate solutions) and directed edges (denoting the resin flow directions). The graph-search capability of Dijkstra’s algorithm is then employed to find the injection configuration that minimises the distance between the gate and the vents without forming dry spots. In another study, Jiang et al. [56] transformed the search space into a Euclidean distance-based graph, greatly simplifying and reducing the search-space size. The aim of the transformed problem is then to find the path that minimises the total resin flow distance while ensuring that the resin flows through all the graph vertices to ensure a thorough mould filling, which essentially becomes the *travelling salesman problem*.

In the literature, most of the LCM mould-configuration optimisation studies that applied problem transformation have reported a significant reduction in the number of optimisation iterations required, although inconsistencies in the solution optimality are observed [8,27,51,54]. It is worth mentioning that capturing an accurate representation of the problem domain is of utmost importance when transforming the search space into an alternative form. The transformed space must host all the critical material and process information correctly defined to provide a consistent, accurate representation of the original problem. It is observed from the literature that, while most of the graphical representation models developed can account for complex material properties (e.g., non-homogeneous/anisotropic reinforcement permeability) [42,53,55], those that rely solely on geometrical distance-based assumptions cannot [8,54,56]. Note that this restriction can be marginally evaded by introducing appropriate process-specific weightings during the construction of the graphical representation, although a massive amount of computational resources will be required for parameter finetuning, potentially invalidating its cost-saving purpose [27,47,51,56]. It is also vital to not oversimplify (or complicate) the problem search space to minimise the likelihood of accidentally omitting the actual global optimum (or a satisfactory local optimum depending on the level of solution optimality desired).

As the problem domain and search space can be represented by various graphs and data structures (e.g., weighted/directed graph, planar graph, path graph, tree, etc.), the appropriate selection of graph (data) structure with respect to the algorithm used is pivotal, and vice-versa [8,16,47,48]. Regrettably, in the context of LCM mould-filling problems, the mathematical/geometrical models of the domain transformation models are onerous and not readily available currently [8,51,53,55]. As a consequence, complementary sets of algorithms need to be developed to generate the transformed search space and integrate it into existing simulation packages, creating technical barriers to adoption as a high level of expertise is required of the practitioner. In addition, these space-transformation models often come with restrictive conditions and prerequisites that may limit their practical utility and ease of implementation, with some examples including the monotonicity condition for BBS and the convexity requirement for CVT [48,53,54,55].

## 5. Search-Space Reduction

The application of search-space reduction, a closely related research field to problem transformation, is also instrumental in minimising the computational cost of simulation-based optimisation. It is worth highlighting that the application of problem transformation typically results in the reduction of search-space size, though the inverse is not true [16,43,46,57]. Aiming at settling for some quick yet satisfactory solutions, search-space reduction refers to the act of restricting the explorable/feasible search space to a smaller subset in an effort to minimise the total amount of solution evaluations required during the simulation-based optimisation process (*ordinal optimisation* [18,19]). Rather than becoming overwhelmed by the total number of possible candidate solutions when searching for the absolute global optimum, it is often beneficial to just sample over a smaller but promising set of good solutions. Some notable search-space reduction techniques in the literature include *factor screening* (i.e., screening out input variables that do not influence the objective function significantly) and *solution screening* (i.e., screening out known/predictable subpar solutions) [16,18,46,57]. Fundamentally, the framework of search-space reduction is inherently different from model-order reduction. In model-order reduction, the process/simulation model itself is simplified, altering the accuracy of the resultant search space. On the contrary, the original process/simulation model is used during search-space reduction (with some candidate solutions ignored during the search process), ensuring solution accuracy. However, the solution optimality is still dependent on the execution of the search-space reduction.

**Figure 7:**
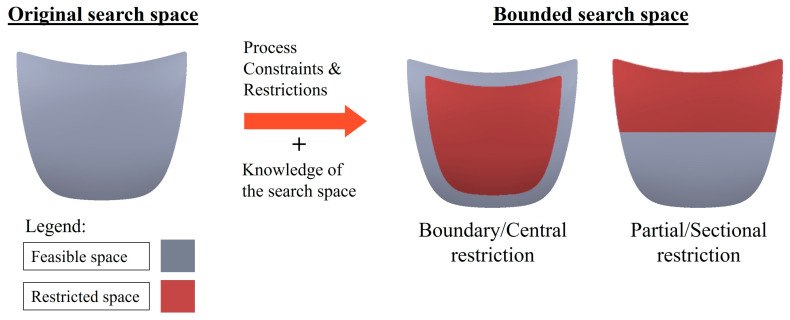
An example of search-space reduction for a car hood model, with the red region indicating the restricted search space excluded from the search process.

Search-space reduction can also be achieved by search-space bounding, where the feasible search space is restricted to only a portion of the total search space. A figurative example of search-space bounding is provided in Figure 7. Search-space bounding is generally performed with respect to the specific process environment and constraints at hand (e.g., mechanical and/or aesthetic requirements, equipment limits, etc.), often with the aid of heuristics [7,16,18,45]. For instance, when searching for the optimal injection location for their in-house RTM moulds, Ye et al. [51] and Gou et al. [58] confined the feasible search space to just the mould boundaries due to restrictions arising from the parts’ aesthetic requirements and their mould-making capability. Search-space bounding can reliably bring massive computational savings in simulation-based optimisation applications, especially if the search space is huge but predictable/monotonous (e.g., mould-filling of reinforcements with homogeneously isotropic permeability). As the cost of performing the solution evaluations (i.e., numerical simulation) is high, it is often uneconomical to exhaustively evaluate all possible candidate solutions within the search space. Rather than blindly sampling from the large set of possible solutions, known domain information and insights of the mould-filling process should be utilised the reduce the search-space size by screening out improbable candidate solutions early and filtering out randomness in searching for better solutions [13,51,57]. For example, research studies have suggested that the central regions of the mould geometry are, in many cases, suitable for placing the injection gates as they will result in the shortest resin flow distance and consequently the least flow resistance [41,59,60]. This known process regularity can be exploited by bounding the search space to the said region and treating it as the sole explorable search space since the likelihood of finding a satisfactory solution is high within that solution subset [8,18,41,54].

**Figure 8:**
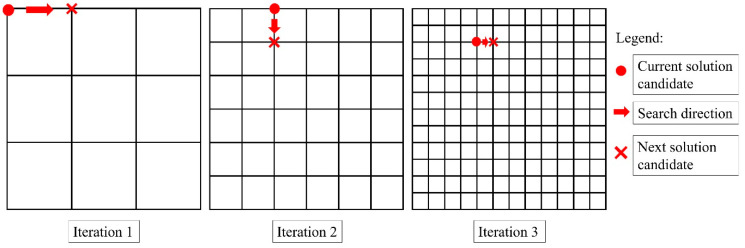
Gradual global mesh refinement across multiple simulation-based optimisation iterations.

**Figure 9:**
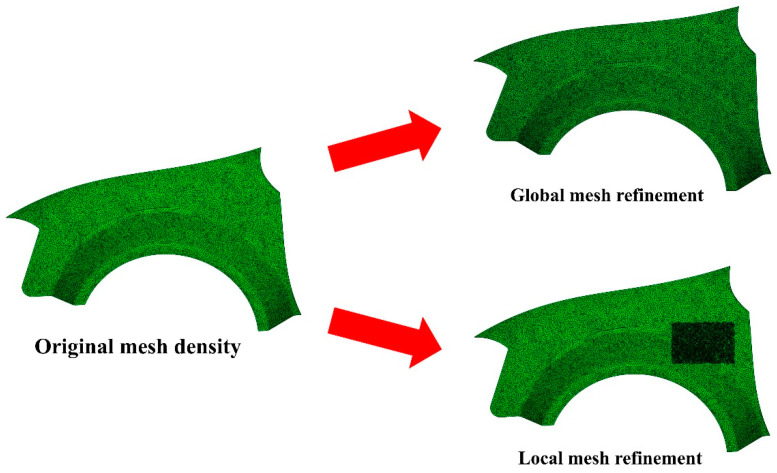
Mesh-density refinement: global mesh-density refinement (entire domain) and local mesh-density refinement (small rectangular area).

Another search-space reduction strategy unique to FE simulation-based optimisation problems is the mesh-density manipulation scheme. As the numerical simulation of the mould-filling process generally operates on the basis of finite element computation, upon domain discretisation, all nodes on the surface of the mould geometry mesh are candidate solutions to the optimisation problem. Thus, the utilisation of a coarser mesh density will directly result in the reduction of search-space size as lesser candidate solutions (i.e., surface nodes) are generated, and the inverse is true. Conveniently, the mesh density adopted during domain discretisation also dictates the process accuracy and the corresponding computational cost [20,23,61,62]. The finer the mesh discretisation, the more accurate and computationally demanding the mould-filling simulation is, and vice-versa [20,62,63]. This cost–accuracy trade-off can also be exploited in tandem to search-space reduction [29,53,62]. To reduce the computational cost whilst maintaining an acceptable level of process accuracy and optimisation reliability, one can initiate the simulation-based optimisation process with simulations of a coarser mesh and progressively refine the mesh size as the search advances throughout the optimisation iterations, as depicted in Figure 8. Smart manipulation of mesh density can bring considerable computational savings in the early optimisation iterations while also allowing the search to converge to the optimum accurately towards the later iterations [23,29,53,56]. Since the candidate solution is, in most cases, far from the optimum during the early optimisation iterations, the overall search performance is unlikely to be impacted, securing a desirable level of process accuracy and optimisation reliability concurrently [7,23,29,49]. This progressive mesh-refinement scheme is felicitous for iterative optimisation algorithms that approach the optimum through local search strategies. It is also worth highlighting that the mesh refinement can be performed either locally (around the current solution candidate) or globally (on the entire meshed domain), as shown in Figure 9. The adoption of local mesh refinement is often crucial to ensure process accuracy while saving cost, as the resin flow behaviour in some regions within the mould (e.g., around the injection location) may be more significant (*resolution-sensitive*) than in other regions [23,29,61,64].

To summarise, the felicitous application of search-space reduction can reliably minimise the cost of simulation-based optimisation while securing a desirable level of solution optimality. Nevertheless, while the strategy allows for a more thorough and quicker search within the reduced search space, search-space reduction may greatly limit solution diversity [16,46,50,51]. In addition, similar to the concepts of problem transformation and the graphical translation of search space, the possibility of accidental omission of probable solutions exists during search-space reduction [10,15,16,46]. It is thus recommended to perform preliminary random samplings around the restricted search region prior to commencing the optimisation process to justify their exclusion. Similar to the application of problem transformation, the adoption of search-space reduction techniques also requires extensive domain-specific knowledge from the practitioner, which may be lacking prior to performing the process or simulation. Their adoption must be carefully considered as it has been reported that problem transformation and search-space reduction techniques may be misleading and may result in specious solutions for highly non-linear problems [44,47,48,50].

## 6. Conclusions

In this paper, potential cost-saving contrivances and cheaper modelling/computational alternatives tailored for LCM mould-configuration optimisation problems are presented and investigated. The comprehensive analysation and assimilation of their operation alongside applicability towards the problem domain of interest are accomplished in this paper to further complement and contribute to future simulation-based optimisation capabilities for composite moulding processes. As emphasised by recent studies across the literature [8,11,21], the integrative research gap fulfilled by this paper is pivotal to promoting and lowering the barrier to the adoption of simulation-based optimisation in industrial settings. Rather than treating the problem as a black-box optimisation problem, problem-specific information and problem domain/landscape knowledge accumulated across the vast literature should be exploited to facilitate the simulation-based optimisation process/framework. The a priori identification of costly modelling approaches and unnecessary wastage of computational resources is crucial to minimise the cost of simulation-based optimisation for LCM processes. For applications requiring a high level of concurrency, for instance, for on-line process monitoring or process digital twinning, the hastening of solution evaluation is much needed. Potential alternatives and simplifications are thus introduced to circumvent, or at least alleviate, the computational challenges discussed earlier.

Nevertheless, most of the contrivances and strategies discussed in this paper minimise the cost of simulation-based optimisation at the expense of process accuracy and potentially solution quality as well. For LCM mould-configuration optimisation problems, this cost-accuracy trade-off is generally permissible as the slight process inaccuracies during the early optimisation stages do not severely invalidate the final optimisation outcome [11,23,29,65]. Additionally, the final solution obtained (or the later iterations nearing solution convergence) can be verified subsequently by a full-order numerical simulation as well. Hence, cost-saving contrivances and strategies that mildly sacrifice process accuracy for efficiency can be adopted at relatively low risk within the early optimisation stages, while the slight inaccuracies are compensated for in the later stages. Nonetheless, a careful compromise between the optimisation cost and output reliability is still of paramount importance [7,11,12,63]. Lastly, akin to the choice of optimisation algorithm, the appropriate adoption of these cost-saving contrivances during simulation-based optimisation is also case-dependent and critical to their effectiveness. If applied in inappropriate scenarios, the cost-saving contrivances and strategies discussed in this paper may diminish, disrupt, or even fail their intended purpose. Hence, each of their specific applicability, efficacy, and suitability towards different optimisation/moulding scenarios are investigated and recapped towards the end of each of their respective sub-sections.

## Data Availability

Not applicable.
